# Key issues in Rett syndrome: emotional, behavioural and autonomic dysregulation (EBAD) - a target for clinical trials

**DOI:** 10.1186/s13023-018-0873-8

**Published:** 2018-07-31

**Authors:** Jatinder Singh, Paramala Santosh

**Affiliations:** 1 Department of Child and Adolescent Psychiatry, Institute of Psychiatry, Psychology and Neuroscience, King’s College London, London, UK; 2 Centre for Interventional Paediatric Psychopharmacology and Rare Diseases, South London and Maudsley NHS Foundation Trust, London, UK

**Keywords:** Emotional, Behavioural and autonomic dysregulation, Rett syndrome, Autonomic dysfunction, Outcome measures, Clinical trials

## Abstract

Complex neurodevelopmental disorders need multi-disciplinary treatment approaches for optimal care. The clinical effectiveness of treatments is limited in patients with rare genetic syndromes with multisystem morbidity. Emotional and behavioural dysregulation is common across many neurodevelopmental disorders. It can manifest in children across multiple diagnostic groups, including those on the autism spectrum and in rare genetic syndromes such as Rett Syndrome (RTT). There is, however a remarkable scarcity in the literature on the impact of the autonomic component on emotional and behavioural regulation in these disorders, and on the longer-term outcomes on disorder burden.

RTT is a debilitating and often life-threatening disorder involving multiple overlapping physiological systems. Autonomic dysregulation otherwise known as dysautonomia is a cardinal feature of RTT characterised by an imbalance between the sympathetic and parasympathetic arms of the autonomic nervous system. Unlocking the autonomic component of emotional and behavioural dysregulation would be central in reducing the impairment seen in patients with RTT. In this vein, Emotional, Behavioural and Autonomic Dysregulation (EBAD) would be a useful construct to target for treatment which could mitigate burden and improve the quality of life of patients.

RTT can be considered as a congenital dysautonomia and because EBAD can give rise to impairments occurring in multiple overlapping physiological systems, understanding these physiological responses arising out of EBAD would be a critical part to consider when planning treatment strategies and improving clinical outcomes in these patients. Biometric guided pharmacological and bio-feedback therapy for the behavioural and emotional aspects of the disorder offers an attracting perspective to manage EBAD in these patients. This can also allow for the stratification of patients into clinical trials and could ultimately help streamline the patient care pathway for optimal outcomes.

The objectives of this review are to emphasise the key issues relating to the management of EBAD in patients with RTT, appraise clinical trials done in RTT from the perspective of autonomic physiology and to discuss the potential of EBAD as a target for clinical trials.

## Background

The Autonomic Nervous System (ANS) can be separated into the sympathetic and parasympathetic nervous system and through multiple overlapping hierarchical networks; these systems continuously orchestrate and fine-tune numerous voluntary and involuntary bodily processes. Any abnormality of the ANS, otherwise known as dysautonomia or autonomic dysregulation, leads to a complex physiological picture. Clinically, autonomic dysregulation presents with a constellation of abnormalities in different components of the sympathetic and parasympathetic nervous system. This leads to imbalances in cardiac, enteric, motor and respiratory systems resulting in an autonomic crisis. There are several medical conditions that are due to imbalances in these systems and some of these include neuroleptic malignant syndrome, malignant hyperthermia, traumatic brain injury and autonomic dysreflexia [1].

At the genetic level, Riley-Day syndrome otherwise known as familial dysautonomia is a rare hereditary autonomic neuropathy caused in the majority of cases by a mutation in the *IKBKAP/ELP1* gene [2]. This gene encodes the protein IKAP that is a crucial component for elongator genes, which are thought to be responsible for the development and maintenance of the ANS [3]. Despite this, the pathophysiology of autonomic dysregulation remains speculative. Studies on the *IKBKAP/ELP1* gene pathway have indicated its role in neurological disorders such as those related to intellectual disability [4], epilepsy [5] and amyotrophic lateral sclerosis [6]. Autonomic failure can also result from other disorders such as those on the autoimmune and neurological spectrum including synucleinopathies, autonomic ganglionopathies and autonomic neuropathies, and have been described in detail elsewhere [7–10]. From a clinical perspective, autonomic dysregulation remains a diffuse entity in that it does not have a standardised and well-defined nomenclature that is widely accepted for clinical practice. So far under its umbrella, more than 30 names have been coined such as central autonomic dysfunction, paroxysmal sympathetic hyperactivity and hypothalamic–midbrain dysregulation syndrome [11].

A cardinal feature of autonomic dysregulation is the disruption of neurotransmitter signalling pathways that can lead to perturbations in the central and peripheral release of neurotransmitters. This can give rise to the manifestations seen clinically and is typically evident in Rett Syndrome (RTT) whereby brainstem immaturity [12–14] leads to underdeveloped neurotransmitter pathways such as those belonging to the serotonergic neurotransmitter system [15, 16]. Others have shown that increased leptin levels in RTT appear to be associated with sympathetic over-activity as evidenced by a significant correlation between plasma leptin levels and the LF/HF ratio (an index of sympatho-vagal balance) [17]. Studies in *MeCP2* null mice have also implicated abnormalities in the locus ceruleus to impaired sympatho-vagal balance [18]. Further research is warranted to confirm these hypotheses.

This review will first introduce autonomic dysfunction and then describe Emotional, Behavioural and Autonomic Dysregulation (EBAD) in patients with RTT. Clinical trials done in patients will RTT will then be placed into context to provide insights that might influence the development of EBAD as a target for clinical trials.

## Aim

The aim of this review is to appraise clinical trials undertaken in patients with RTT from the perspective of autonomic dysregulation.

## Method

For the purposes of this review freely available biomedical databases PubMed, Cochrane and Scopus were searched. So that the search could be made more relevant, the Boolean Operator ‘AND’ was used to link the search terms together. The search words were ‘Rett Syndrome AND Clinical Trial.’ To improve the validity of this search, the following inclusion and exclusion criteria were implemented into the search strategy:

### Inclusion criteria

- Articles in English language in academic journals.
- Peer reviewed articles.
- Articles available electronically.

### Exclusion criteria

- Reviews, conference papers, short surveys, notes, book chapters, editorials, letters and articles in press.
- Articles not in English language.
- Articles not readily accessible electronically.

**Fig. 1:**
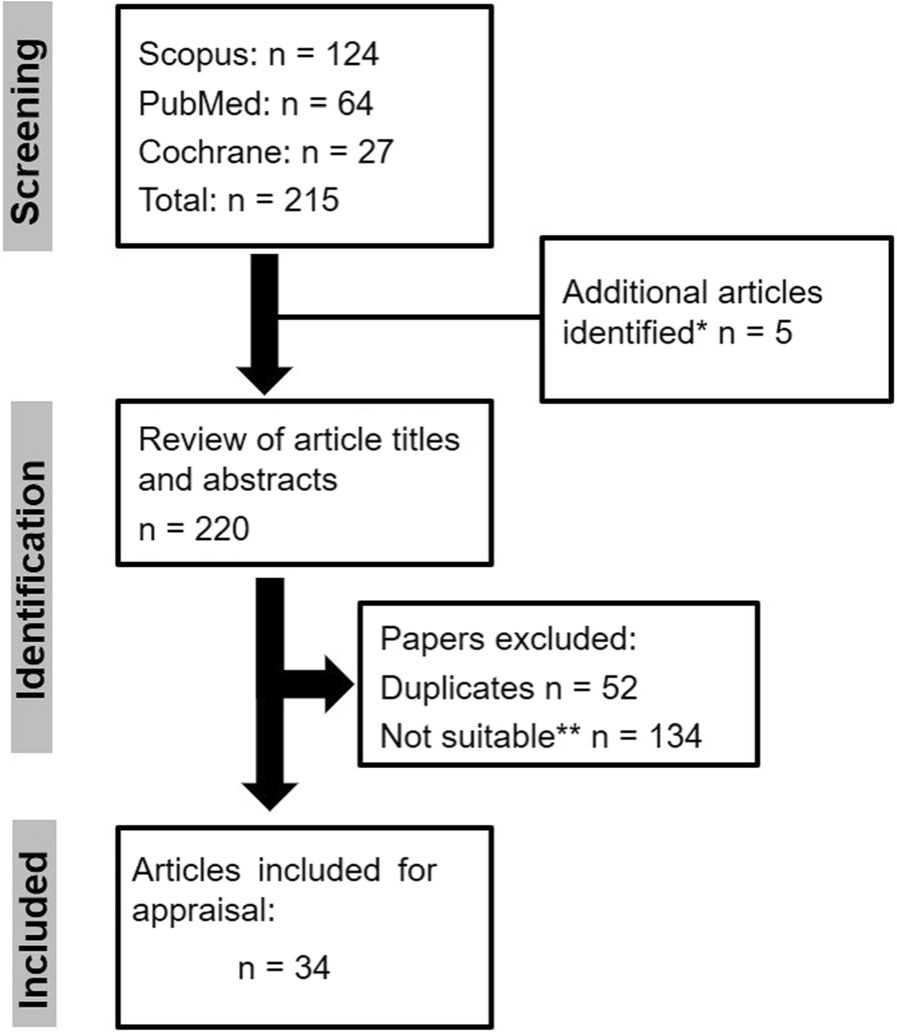
Screening process and search results. Notes: * Yuge et al. 2017 (reference: 129); Pini et al. 2016 (reference: 125); Pini et al. 2012 (reference: 119); Signorini et al. 2011 (reference: 115); Haas et al.1986 (reference: 101). ** Based on inclusion/exclusion criteria. Studies done in animal models, review articles and irrelevant articles were excluded

The search strategy showing the information about data sources, screening, identification of studies and the number of studies included is described in Fig. 1.

## Autonomic dysfunction in Rett syndrome

### Neuronal vulnerability in Rett syndrome

Having an incidence of approximately 1:10,000 live births [19], RTT is a debilitating neurodevelopmental disorder predominantly observed in females. In the vast majority of cases (~ 90%), sporadic functional loss of the *methyl-CpG binding protein 2* (*MeCP2*) gene causes RTT, with mutations in *CDKL5*, *FOXG1*, *CTNNB1* and *WDR45* genes [20–22] contributing to the rarer atypical or variant RTT phenotypes. A strong evidence base points towards the MeCP2 protein being an epigenetic modulator [23–26] with pleiotropic properties binding to more than 40 structurally diverse proteins [27]. Consistent with its role as a modulator of chromatin architecture [28] more recently it was shown that the overarching function of MeCP2 was to recruit the NCoR/SMRT co-repressor complex to methylated sites on chromatin [29]. By connecting DNA to the NCoR/SMRT complex, it is likely that the *MeCP2* gene has far reaching genome level properties that have a critical role on the impact of genes regulating pre- and post-natal neuronal development in either the upregulation of long (> 100 kb) [30] or short genes [31]. With a wide body of evidence indicating that modulation of epigenetic mechanisms associated with gene length are associated with neurodevelopment disorders [32], it underscores the premise that disruption of gene length represents a major molecular locus of vulnerability for neurons in RTT. This neuronal vulnerability can manifest as altered neurogenesis, migration and synaptic integration, and clinically can present itself as impaired developmental, motor, and social skills.

### Autonomic dysfunction

The clinical phenotype of RTT is broad. Being an X-linked gene, there are marked differences in the expression of wild-type and mutant alleles [33, 34], which has an impact on the degree of functional impairment seen in patients [35]. In terms of the degree of functional impairment, at present autonomic dysfunction appears not to be governed by a specific mutation unlike motor or cardio-respiratory phenotypes [35, 36]. Despite this, in patients with RTT the incidence of autonomic dysregulation is very high (~ 75%) [37] and is regarded to be a key driver of sudden death [38].

The fluidity by which MeCP2 manipulates neuronal function bestows on it a unique feature that has made the precise pathophysiology of the autonomic dysregulation seen in patients with RTT difficult to understand. Nevertheless, autonomic dysregulation has been explored in patients with RTT [17, 39–46]. One hypothesis is that unrestrained vagal tone causes a sympatho-vagal imbalance that is considered unique to patients with RTT [36]. Sympatho-vagal imbalance might contribute to about ¼ of the sudden deaths observed in females with RTT [47, 48], although these numbers are probably overestimated given the incomplete reporting of the cause of death in more recent studies [49, 50].

### Targeting the clinical phenotype

The clinical phenotype of autonomic dysregulation is highly variable as individuals can exhibit broad inter-individual differences. In RTT, the autonomic dysregulation can overlap between the sympathetic, parasympathetic and enteric nervous system. Clinically, patients can present with generalised anxiety, panic attacks, breathing dysfunction, temperature dysregulation, peripheral vascular changes, enteric changes and cardiac abnormalities, and treatment often requires a personalised approach. Current strategies are largely directed towards normalising the symptoms of autonomic dysregulation in particular modifying the deleterious cardio-respiratory phenotype, which not only has a significant impact on disorder burden [51] but also seems to be the most viable clinical outcome measure for translation [52]. Targeting the serotonergic neurotransmitter system therefore appears to be a viable symptomatic strategy [53, 54], and presently studies are underway exploring the role of a 5-HT1A receptor agonist for the reduction of respiratory impairments in females with RTT [55]. More recently, autonomic dysregulation due to altered levels of Substance P expression in the brainstem of *MeCP2* null mice was also found to be an associative factor for the respiratory deficits seen in these mice [56]; however, further work would be warranted in human studies before inferences can be made.

## Emotional and Behavioural dysregulation

### The interplay between emotion and behaviour

The ANS is the mainstay for the synergistic interplay governing human emotion and behaviour. In particular, the parasympathetic feedback of cardiac tissue via the vagus nerve provides a means for cardiac-respiratory output to be regulated to meet ever-changing emotional and behavioural demands [57]. Disorders in this feedback pathway and allied interconnecting networks belonging to the anterior cingulate cortex [58] and other brain regions such as the amygdala [59] are thought to be key drivers for emotional and behavioural dysregulation seen in a wide range of developmental disorders. From a developmental perspective, emotional dysregulation in childhood is associated with varied psychiatric and psychosocial deficits in adolescents [60]. Moreover, a 14-year prospective follow-up study in 2076 children showed that emotional dysregulation in childhood confers an increased risk of emotional dysregulation in early adulthood [61]. It is likely that developmental history influences the malleability of brain networks and therefore epigenetic factors associated with childhood adversity or maltreatment can have a marked impact on the developmental trajectory of behavioural and emotional brain conduits propagated into adulthood. Indeed, evidence points towards altered brain networks such as changes in the threshold of limbic reactivity in response to early childhood adversity [62] and disrupted fronto-limbic circuits as the most altered brain regions in those who have experienced childhood maltreatment [63]. These circuit changes are also consistent with the premise that these children are at higher risk of reactive aggression and autonomic hypo-responsivity [64, 65]. Developmental traumatology is also believed to alter stress hormone responses that in turn can modify neuronal morphology in brain regions resulting in functional perturbations such as decreased right and left hemisphere integration [66]. In RTT, neuronal vulnerability and the increased electrical irritability of neural circuits produce marked changes in emotion and behaviour, and the salient points will be discussed below.

### Emotional and Behavioural dysregulation in RTT

Emotional and behavioural dysregulation is frequently encountered in patients with RTT [51, 67, 68]. In RTT, the emotional state of the individual can be further exacerbated by the physical difficulties observed, for example, epileptic seizures can lead to a heightened emotional state and often leads to anxiety [69] but can also include screaming, labile mood and uncontrollable crying [67, 70]. Regarding lifespan, emotion and behaviour are thought to change during the time course of RTT [71, 72]. Behavioural dysregulation can present with increased stereotypies, repetitive rocking, scratching, self-injurious or self-stimulatory behaviour, and agitation. Some studies have indicated that emotional and behavioural impairments such as sleep problems and screaming in the early stages of RTT may be due to the occurrence of intellectual disability rather than by RTT itself [73, 74] and may reduce over time [37, 75]. In RTT, evidence has shown that individuals with milder mutations are more likely to exhibit mood disturbances such as anxiety/inappropriate fear in comparison to individuals with more severe mutations who were less likely to report such difficulties [76].

While the precise cause of the emotional and behavioural dysregulation in RTT remains to be established, developmental deletion of *MeCP2* gene in somatosensory neurons in animal models can recapitulate the core behaviour defects observed in RTT such as anxiety [77]. Recently it was also shown that MeCP2 could restrain corticotrophin releasing hormone gene expression [78], which has a key role in maintaining the homeostasis of the hypothalamic-pituitary-adrenal axis (HPA). Dysregulation of the HPA has been implicated in a variety of childhood anxiety disorders [79].

Emotional and behaviour dysregulation can have a significant impact on the quality of life in patients with RTT. Despite this, the association of autonomic dysregulation on emotional and behavioural dysregulation in RTT has not been well developed. The triumvirate consisting of (I) emotion, (II) behaviour and (III) autonomic function needs to be considered holistically in RTT and will be discussed in the next section.

## Emotional, Behavioural and Autonomic dysregulation in RTT

Given the nature of the disorder, the emotional and behavioural dysregulation seen in patients with RTT has been difficult to capture. Most of the information relating to the emotional and behavioural state has been based on proxy measures such as the motor–behavioural assessment (MBA) [80], the Rett Syndrome Behavioral Questionnaire (RSBQ) [81], Anxiety Depression and Mood Scale (ADAMS) [82], the Vineland Adaptive Behaviours Scale [83], the Rett Clinical Severity Score (RCSS) [84] and the Gross Motor Scale [85]. Quality of life measures such as the Child Health Questionnaire-P50 have also been used [86]. Others have attempted to use direct observation to recognize the emotional state in patients with RTT [87, 88]; however, the outcome of the emotional and behavioural state by observers is poorly defined with no consensus on agreement by observers and underscores the need for an individualised approach.

**Fig. 2:**
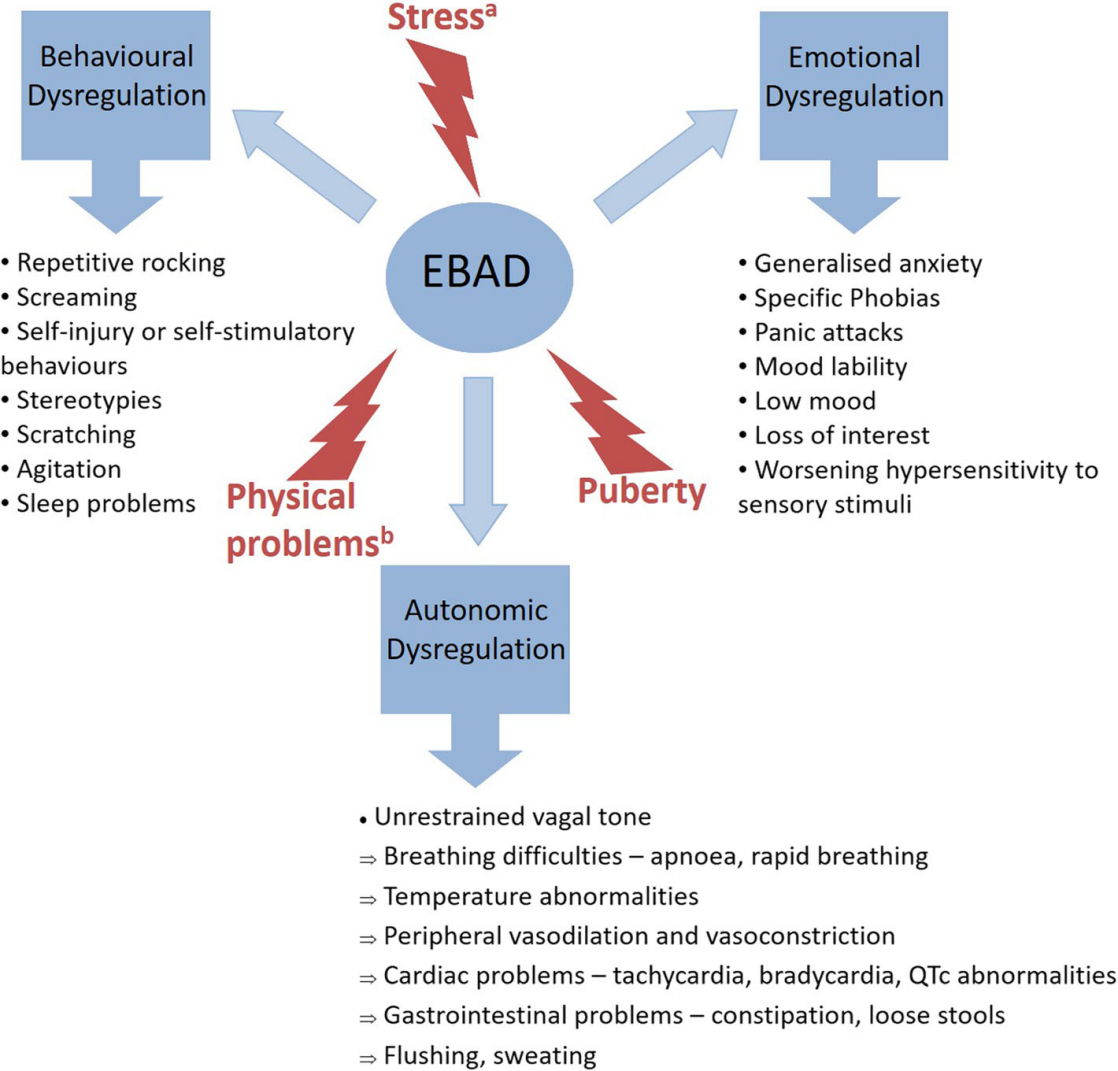
Emotional, Behavioural and Autonomic Dysregulation (EBAD) and its clinical presentation in Rett Syndrome. Abbreviations: EBAD (Emotional, Behavioural and Autonomic Dysregulation). Notes: ^a^ Stress can cause functional changes in epigenetics and it is highly probable that the epigenetic mechanisms controlled by *MeCP2* form a crucial component of the stress response. ^b^ Physical problems include seizures, pain and discomfort, and infections

Autonomic function together with emotional and behavioural regulation is of interest when capturing clinically meaningful change longitudinally in patients with RTT. The symptoms of EBAD and how they present clinically is shown in Fig. 2. To manage this symptomatology, biometric guided therapy can be used to map the trajectory of EBAD in patients with RTT, and in those with a significant functional disability. Heart rate variability (HRV) is an indirect measure of autonomic arousal [89, 90] and can be measured using wearable sensor technology. Wearable sensor technology has been used as a biometric proxy measure to monitor treatment outcomes of EBAD in RTT [40, 91] and in those with other complex neurodevelopmental disorders [92]. In these cases, sensor-based biometrics can assist in the individualisation of patient care allowing the management of patients with EBAD. This strategy is currently being used in the Centre for Interventional Paediatric Psychopharmacology and Rare Diseases (CIPPRD) [93]. Further studies are warranted in other routine clinical settings.

Given that EBAD is seen in treatment non-responders with a significant functional disability frequently encountered in rare diseases and in those with complex neuropsychiatric problems, there is an urgency to provide effective treatment pathways for these patients. Despite this, there is a formal lack of guidelines on the management of EBAD in patients with RTT and highlights the need for improvements in knowledge within this area in particular regarding clinical trials. While RTT specific anchors [94] and Bayesian approaches [95] have improved outcome measures and the design of rare disease clinical trials, the ultimate goal is to harmonise interventions that can be used to monitor EBAD in RTT patients across clinical trials. Autonomic data can help in this regard and might be able to foster the development of better outcome measures for clinical trials as was shown recently in patients with breathing abnormalities [96]. Similarly, in a cohort of RTT patients, biometric data (electrodermal activity and HRV) has been used to detect an autonomic response to pain [97]. In the next section, the potential utility of EBAD as a target in clinical intervention trials will be discussed.

## EBAD as a target for clinical trials in RTT

Given the clinical heterogeneity observed in patients with RTT, there is unlikely to be a ‘one-size-fits-all’ treatment approach. Disentangling the heterogeneity of RTT across individuals has been a real treatment challenge in terms of providing effective clinical interventions. Traditionally, such an approach often requires the use of ‘big data’ to identify patterns of interaction seen across individuals, and although big data analytics are gaining traction in psychiatry [98], such approaches are not readily feasible in RTT given the limited patient population and the difficulties in assessing patients during the life-span of the disorder. This has been exemplified in individuals with Fragile X syndrome (FXS). As a single gene disorder, FXS was thought to have had amenable drug targets, however, despite promising studies using animal models, so far, the translational potential of drug treatment for FXS has failed to live up to expectation even using well-powered double-blind placebo-controlled trials [99]. Failure of drug translatability has prompted the re-assessment of outcome measures in FXS [100] and underscores the need for more objective measures for clinical trials in RTT patients that would be able to capture clinically meaningful change longitudinally.

### Clinical trials in Rett syndrome

From the search strategy (Fig. 1), 34 articles (references: [101–134]) were identified and appraised. These studies are summarised in Table 1. Details relating to the different domains of EBAD i.e., the emotional, behavioural and autonomic components have been emphasised in Table 1. Although none of the studies specifically used EBAD as a target for clinical trial intervention, a few did show promise in improving the indices of EBAD reflected in improvements in emotional, behavioural and/or autonomic dysregulation.

**Table 1:** Clinical trials involving RTT patients with commentary relating to the specific domains of EBAD

Authors	N^a^	Duration (months)	Design	Intervention	Physiological Measurement(s) of Autonomic Function	Outcome Measures	Comments#	Reference
Mancini et al. (2018)	36 (26)	6	Randomised multicentre, placebo controlled double-blind	Desipramine	• ECG • AHI polysomnography • Respiration rate • Oxygen desaturation	Primary: • Change in AHI at 6 months from baseline Secondary: • CSS • SSI • Number of apnoeas • Number of hypopneas • Oxygen desaturation	No significant differences between groups (high dose, low dose or placebo) were observed in the outcome measures (AHI, breathing patterns, CSS or SSI) from baseline to 6 months.	[134]
Downs et al. (2018)	12	6^b^	Modified stepped wedge individually randomised controlled trial	Environmental enrichment	N/A	Primary: • Change from baseline in RSGMS Secondary: • Blood levels of BDNF protein • BMI • Sleep quality as measured using the DIMS subscale of the parent rated SDSC. • Mood as measured using the mood subscale of the RSBQ	• After 6 months of treatment, the enriched environment accounted for improvements in gross motor skills and elevated blood BDNF levels. • No change from baseline in BMI, sleep quality or mood.	[133]
O’Leary et al. (2018)	30 (29)	18^c^	Randomised placebo controlled double-blind crossover	IGF-1 (full length)	Bioradio	Primary: • ADAMS • SA subscale • RSBQ • F/A subscale • PTSVAS top three concerns • CGI • PGI • Kerr (overall severity scale) Secondary: • Additional ADAMS subscales • additional RSBQ subscales • ABC-C subscales • MSEL • Vineland-II • CSBS-DP • Cardiorespiratory biomarkers	No significant improvements were noted between treatment groups	[132]
Glaze et al. (2017)	56^d^	0.5–1	Randomised Phase 2, placebo controlled double-blind	Trofinetide (IGF-1 tripeptide analogue)	• EEG • Electromyography • Oxygen desaturation • HRV • Respiratory rate variability	• Modified apnoea index score • ABC-C • MBA • RSSS • CGI-I • VAS	• Efficacy was demonstrated for trofinetide (Day 26 for 70 mg/kg treatment group, *n* = 17) in comparison to placebo for core features. • Effect sizes (with CI): ➢ CGI-I: − 0.554 (− 1.34, 0.23) ➢ Caregiver Top 3 Concerns: − 0.63 (− 1.42, 0.16) ➢ MBA change index: − 0.607 (− 1.39, 0.18)	[131]
Smith-Hicks et al. (2017)	38^e^	6	Randomised open-label	Dextromethorphan	EEG	• Cognitive status: ➢ MSEL ➢ VABS • Behavioural characterisation: ➢ Parent-completed ABC–Community Version ➢ *SSI ➢ RSSS • Gait • EEG	Dose dependent improvements deemed to be of statistical significance were noted for clinical seizures, receptive language and behavioural hyperactivity; however, there was no statistically significant improvement in global severity as assessed by the RSSS.	[130]
Yuge et al. 2017	4	10-24^f^	Pilot open-label	Ghrelin	Not measured	• SDCF to assess clinical and neurological parameters • BFMDRS score to assess dystonia • VAS	Following treatment, improvement in scores were noted for: • SDCF for Patient 1 (31 [baseline] to 29 [2 years]) and Patient 2 (20 [baseline] to 18 [10 months]) • BFMDRS for Patient 1 (108.5 [baseline] to 100 [2 years]) and Patient 2 (67 [baseline] to 54.5 [10 months]) • Change in baseline in VAS for dystonia, tremor and sympathetic vasomotor reflexes in patient 1 and 2.	[129]
Nissenkorn et al. 2017	14	6 (planned)	Open-label (Phase 2)	Glatiramer Acetate	Noninvasive respiratory inductance plethysmography	Primary: • Safety, tolerability of treatment • Decrease in epileptiform activity Secondary: • Seizure frequency • Improvement in respiratory dysfunction • Improvement in core features assessed by Kerr and Naidu validated severity scores)	Study was terminated due to treatment related serious adverse events in four patients.	[128]
Djukic et al. (2016)	10	6	Open-label (Phase 2)	Glatiramer Acetate	• Xltek, Sleep Monitoring • Tobii T300 Eye Tracker • EEG	Primary: • Change in gait velocity (primary) Secondary: • Change in respiratory and cognitive functions, • Change in QOL measures (assessed using Child Health Questionnaire-P50) and • Change in EEG parameters	No statistically significant changes in QOL were observed, however, following glatiramer acetate treatment, improvements were noted for: • Gait velocity (13–95% improvement [*p* = 0.03]) • Memory and breath holding index (*p* ≤ 0.03)	[127]
Fabio et al. (2016)	34^g^	N/A	Observational	Cognitive training	• Eye-tracking • EEG	Functional and cognitive descriptive of sample using functional scales, matrices, eye tracking and EEG assessment.	Longer term (5 days) cognitive training appears to improve behaviour and brain parameters in RTT patients.	[126]
Pini et al. (2016)	10	6	Open-label	IGF-1 (full length)	EEG	• ISS • RSS	Significant improvements seen in ISS (*p* = 0.0106) and RSS (*p* = 0.0274) outcome measures for the treatment group.	[125]
Khwaja et al. (2014)	12	1 MAD 5 OLE^h^	Open-label (Phase 1)	IGF-1 (full length)	• BioRadio • EEG	• Apnoea Index • Hyperventilation Index • MBA • RSBQ • ADAMS • EEGs	Following IGF-1 treatment, improvements were noted for: • Apnoea from Pre-MAD to Post-OLE (Apnoea index −7.12 ± 4.58 [mean ± SE]) • RSBQ (fear/anxiety subscale: − 0.79 [mean difference from visit 1 of OLE to visit 5 of OLE]), • ADAMS (social avoidance subscale: − 1.44 [mean difference from visit 1 of OLE to visit 5 of OLE]), • Reversal of right-sided alpha band frontal EEG symmetry	[124]
Signorini et al. (2014)	24^i^	12	Randomised, controlled study	ω-3 PUFAs	N/A	Examination of erythrocyte fatty acid profile	Improvements seen in ω-6/ω-3 ratio, serum lipid profiles as well as normalisation of inflammatory markers and reduction in bone hypodensity and PUFS peroxidation following ω-3 PUFA supplementation compared to the untreated group.	[123]
Maffei et al. (2014)	66^j^	12	Randomised, placebo controlled single-blind	ω-3 PUFAs	Echocardiography	Measurement of oxidative stress biomarkers and ECG parameters	Improvements were noted in biventricular myocardial systolic parameters following ω-3 PUFAs treatment in comparison to placebo.	[122]
Hagebeuk et al. (2013)	10	26	Randomised, placebo controlled, double-blind crossover	Folinic acid	N/A	Measurement of CSF, folate metabolite and SAH/SAM ratio	N/A	[121]
De Felice et al. (2012)	20	6	Randomised, placebo controlled single-blind	ω-3 PUFAs	Respiratory polygraphy using Somnowatch	Primary • CSS • Respiratory dysfunction • Video clips pre-& post treatment Secondary • Reduction in oxidative stress markers following treatment	Although respiratory dysfunction improved after 6 months of treatment no significant changes were detected in autonomic symptoms as assessed by the CSS.	[120]
Pini et al. (2012)	6	6	Open-label pilot	IGF-1 (tripeptide form)	• Neuroscope • ECG • EEG	• Autonomic parameters evaluated were cardiac vagal tone, HR, transcutaneous blood gases, and trends in respiration • ISS parameters (growth and development, locomotor apparatus, locomotor ability, cortical and autonomic functions) • EEG measurements	Safety and tolerability study – no statistically significant changes were reported in cardiac function (ECG, HR and vagal tone) or in all ISS parameters during IGF-1 treatment.	[119]
Hagebeuk et al. (2012)	8	26	Randomised, placebo controlled double-blind crossover	Folinic acid	N/A	• Plasma folate measurement • MBA • Hand Apraxia Scale • Parental Overall Well-Being Index	No statistically significant differences were found for neurological features, Hand Apraxia Scale or the MBA	[118]
Hagebeuk et al. (2011)	12	24	Randomised, placebo controlled, double-blind crossover	Folinic acid	EEG	Change in seizure frequency and EEG	Benefits only noted in 3 patients on folinic acid.	[117]
Freilinger et al. (2011)	21^k^	13	Double-blind, randomised, placebo-controlled crossover	Creatine	N/A	• Change in global DNA methylation • Change in RTT specific symptom score as defined by MBA	No statistically significant changes in either the total or sub-scores of the MBA	[116]
Signorini et al. (2011)	42^l^	12	Open-label pilot	ω-3 PUFAs	N/A	Measurement of oxidative stress biomarkers	N/A	[115]
Leoncini et al. (2011)	42	12	Open-label pilot	ω-3 PUFAs	N/A	Measurement of oxidative stress biomarkers	N/A	[114]
Temudo et al. (2009)	25	6	Open-label in patients with low levels of folate metabolite	Folinic acid	N/A	Measurement of folate metabolites and CSF neurotransmitters	N/A	[113]
Glaze et al. (2009)	73 (68)	12	Randomised double-blind, placebo-controlled	Folate–betaine	Polygraphic measurements of breathing patterns, hand stereotypies and qualitative EEG	• Growth parameters (weight, height, BMI, and head circumference • EEG • MBA • Parent questionnaire	No objective improvements reported, however, subjective improvement based on a parent questionnaire was noted for the < 5 years age group.	[112]
Wilfong & Schultz (2006)	7	12	Case series	Adjunctive vagus nerve stimulation (VNS) for treatment of epilepsy	Vagus nerve stimulation device	• Seizure frequency • Effect of VNS therapy on caregiver reported hyperventilation, breath holding, swallowing dysfunction, mood and communication.	• Improvements were noted in seizure frequency and in alertness. • No significant change in either mood or communication parameters following VNS therapy.	[111]
Guideri et al. (2005)	10^m^	6–18 months (follow-up)	Randomised blinded study	Acetyl-L-carnitine	ECG parameters	• Heart rate variability • QTc interval • QTc dispersion	Increased heart rate variability was observed in the treatment group.	[110]
Gorbachevskaya et al. (2001)	9	20–40 days^n^	Open-label pilot	Cerebrolysin	EEG	Quantitative EEG to monitor motor and cortical functions.	Modest improvements in motor and higher cortical functions in patients treated with cerebrolysin.	[109]
Ellaway et al. (2001)	21^o^	6	Open-label	L-carnitine	N/A	• RS: SSI • Hand Apraxia Scale • 7 day-night sleep diary • SF-36 Health Survey	Improvements noted in sleep efficiency (*P* = 0.027), expressive speech *(P* = 0.011), communication skills (*P* = 0.004) and energy levels (*P* < 0.005) in the treatment group in comparison to the control group.	[108]
Ellaway et al. (1999)	35	6	Randomised placebo controlled double-blind crossover	L-carnitine	N/A	• MBA • Hand Apraxia Scale • Patient Well-Being Index	• No measurement of autonomic function. • Small improvements in patients’ well-being and hand apraxia scale in the L-carnitine treatment group.	[107]
McArthur and Budden (1998)	9	2.5	Randomised placebo controlled double-blind crossover	Melatonin	Actigraphy (measures of sleep parameters)	Sleep parameters	• Melatonin decreased sleep onset (19.1 ± 5.3 min [mean ± SE]) in comparison to baseline (42.1 ± 12.0 min [mean ± SE]). • Other improvements were noted in total sleep time and sleep efficiency.	[106]
Stenbom et al. (1998)	12	~ 3-5^p^	Open-label pilot	Lamotrigine	EEG	• Seizure frequency • Motor skills • Safety and tolerability monitoring	Some improvements were noted in with lamotrigine regarding seizure frequency, alertness and concentration.	[105]
Percy et al. (1994)	25^q^	9	Randomised placebo controlled double-blind crossover	Naltrexone	EEG polygraphy that assessed sleep, respiratory characteristics and hand stereotypies.	• Neurophysiological parameters (assessment of sleep, respiratory characteristics and hand stereotypies). • Measurement of CSF and β endorphin	• Statistically significant differences were noted for a higher awake minimum O_2_ saturation (P = 0.03) and less time spent on disordered breathing (*P* = 0.02) in the naltrexone treatment group in comparison to the placebo treatment group. • No changes in EEG and sleep characteristics between the treatment and placebo group.	[104]
Nielsen et al. (1990)	11^r^	~ 6	Randomised double-blind crossover	Tyrosine and tryptophan	EEG	• Parent interviews and observation forms to assess child behaviour.	No clinical improvement.	[103]
Zappella (1990)	10	4	Placebo controlled double-blind partial crossover	Bromocriptine	N/A	• Portage guide items for the assessment of motor, social and cognitive skills.	• In the treatment group, improvements in Portage guide items for motor, social and cognitive activities were noted for 2 subjects and minor improvement in 1 subject. • 7 subjects showed no change to treatment.	[102]
Haas et al. (1986)	7	2–6	Open-label uncontrolled treatment trial	Ketogenic diet	• EEG • Transthoracic impedance to assess respiratory characteristics (central apnoea).	Changes in: • EEG • Respiratory monitoring • Clinical laboratory values	Clinical improvements noted in: • Seizure control, • Respiratory function, • Behavioural and motor control.	[101]

*Abbreviations*: *ABC-C* Aberrant Behaviour Checklist-Community, *ADAMS* Anxiety Depression and Mood Scale, *AHI* Apnoea Hypopnea Index, *BDNF* Brain Derived Neurotrophic Factor, *BFMDRS* Burke-Fahn-Marsden Dystonia Rating Scale, *BID* bis in die - twice daily, *BMI* Body Mass Index, *CGI-I* Clinical Global Impression-Improvement, *CI* Confidence Interval, *CSBS-DP* Communication and Symbolic Behaviour Scales - Developmental Profile, *CSF* Cerebrospinal fluid, *CSS* Clinical Severity Score, *DIMS* Disorders of Initiating and Maintaining Sleep, *EBAD* Emotional, Behavioural and Autonomic Dysregulation, *ECG* electrocardiogram, *EEG* electroencephalogram, *F/A subscale* Fear/Anxiety subscale, *HR* Heart Rate, *HRV* Heart Rate Variability, *IGF-1* Insulin-like Growth Factor 1, *ISS* International Severity Scale also sometimes known as the International Scoring System, *MAD* Multiple Ascending Dose, *MBA* Motor Behavioural Assessment, *MSEL* Mullen Scales of Early Learning, *N/A* not applicable, *OLE* Open-label Extension, *ω-3 PUFAs* omega-3 polyunsaturated fatty acids, *PGI* Parent Global Impression, *PTSVAS* Parent Target Symptom Visual Analog Scale, *RS: SSI* Rett Syndrome: Symptom Severity Index, *RTT* Rett Syndrome, *RSS* Rett Severity Scale, *RSBQ* Rett Syndrome Behaviour Questionnaire, *RSGMS* Rett Syndrome Gross Motor Scale, *RSSS* Rett Syndrome Severity Scale, *SAM* S-adenosylmethionine, *SA subscale* Social Avoidance subscale, *SAH* S-adenosylhomocysteine, *SDCF* Scoring for Different Clinical Features, *SDSC* Sleep Disturbance Scale for Children, *SE* Standard Error, *SF-36* Short Form Survey 36-items, *SSI Screen for Social Interaction, *SSI* Severity Score Index, *VAS* Visual Analog Scale, *VABS* Vineland Adaptive Behaviour Scales, *Vineland-II* Vineland Adaptive Behaviour Scales-Second Edition, *VNS* Vagus Nerve Stimulation

# When applicable comments relate to domains of EBAD specifically commentary on autonomic, emotional and behavioural outcome measures from the studies

^a^Number in parenthesis reflects those participants who completed the study

^b^Intervention period

^c^The study consisted of two treatment periods. Eligible patients were randomly assigned to receive either placebo or active in treatment period 1 (20 weeks) and crossed over to treatment period 2 (20 weeks). Treatment periods 1 and 2 were separated by a 28 week washout period. At the end of treatment period 2, participants had a 4-week follow-up period

^d^Cohort 0 (trofinetide 35 mg/kg or placebo bid, *n* = 9), Cohort 1 (trofinetide 35 mg/kg or placebo bid, *n* = 18) and Cohort 2 (trofinetide 75 mg/kg or placebo bid, *n* = 29)

^e^Thirty eight (38) individuals were randomised and 32 were used for analysis

^f^Intravenous (iv) ghrelin was administered qd (3 μg/kg for 5 min) for 3 days. Patients 1 and 2 who presented with dystonia where administered the same dose of iv ghrelin over 2 days for a period of 3 weeks. In these patients, neurological examinations were performed at 24 months for Patient 1 and 10 months for Patient 2

^g^Twenty one (21) girls underwent training. The control group (did not undergo training) consisted of 13 patients

^h^12 subjects participated in the 4-week MAD study and 10 of these continued and completed the 20 week OLE

^i^Treated *n* = 12, untreated n = 12

^j^Treated *n* = 33, untreated n = 33

^k^Of which 18 were analysed

^l^In the main study 102 patients (reference: [115]) or 113 patients (reference: [114]) with RTT were included. A different cohort of 42 patients was included in the open-label pilot supplementation study

^m^Active treatment group consisted of 10 girls with RTT and was compared to an untreated (control) RTT group of 12 patients

^n^Each course of cerebrolysin therapy consisted of 20 days. Of the nine RTT patients, seven received one course and two received two courses

^o^Also included a control group of 62 RTT patients

^p^Duration of dosage of lamotrigine was individualised depending upon concomitant anti-epileptic drug use. Dosing was terminated between the 17th - 20th weeks in the epilepsy group and between 9th - 12th weeks in the motor group

^q^Of which 22 completed the first treatment period

^r^Nine patients participated in an open-label trial where they received 0.3 g tyrosine and 0.1.g tryptophan/kg body weight for 2–17 weeks. Based on the findings from the open-label study, 11 girls participated in the double-blind crossover trial, for two periods of 8–10 weeks receiving active or placebo. The two treatments periods were separated by the 4-week washout period

Autonomic dysfunction reflected by impaired cardiac output improved following treatment with omega-3 polyunsaturated fatty acids (ω-3 PUFAs). In this study, subclinical myocardial dysfunction was shown to be partially rescued in patients with RTT by high dose ω-3 PUFAs supplementation for 1 year [122].

Other studies paint a complex picture. Insulin-like Growth Factor 1 (IGF-1) in its tripeptide form had no effect on cardiac vagal tone in a study of 6 RTT patients [119]; however, a later study using full length IGF-1 (also known as mecasermin) in 12 RTT patients showed improvements in breathing and peripheral autonomic function but no change in communication or motor functions [124]. An open-label study evaluating the effects of mecasermin in 10 patients also showed improvements in ISS scores [125]. The International Scoring System (ISS) is used to assess RTT severity using five separate subscales one of which considers the brainstem-autonomic component (subscale v). Despite some studies showing positive findings, a recent double-blind crossover trial assessing efficacy of mecasermin in 29 patients noted no significant improvements between treatment groups in the outcome measures tested [132]. Moreover, in that study a secondary analysis of subjects not in the placebo arm showed a worsening of symptoms.

Improvements in gait velocity and breath holding [127] were observed in patients administered glatiramer acetate (GA). On the contrary, a similarly designed study using GA was prematurely terminated due to serious adverse events (SAE) in four patients [128]. The onset of these SAEs followed administration of GA and the subsequent immediate post-injection reaction was deemed to be related to primary autonomic dysfunction [128].

Ghrelin has an important role in maintaining the balance of the parasympathetic and sympathetic nervous system [135]. In a recent pilot study, two patients with RTT showed improvements in dystonia, tremor and vasomotor reflexes following treatment with ghrelin [129]. It is possible that these improvements in clinical symptoms may be due to the ghrelin dampening down the hypertonic state of the sympathetic arm of the nervous system; however, further work would be needed in a larger sample size and randomised controls to confirm this finding.

Using a variety of outcome measures, improvements were noted in the core clinical features following dextromethorphan [130] and high dose trofinetide (IGF-1 tripeptide analogue) [131] treatment in RTT patients. Interestingly, in these studies not all parameters that were evaluated demonstrated improvements. No changes in either global severity [130] or the apnoea index [131] were noted in comparison to placebo. This was shown in a study of desipramine in 26 patients with RTT whereby no significant differences were found between treatment groups for apnoea-hypopnea index and severity [134].

The apparent contradictory findings in some of the studies previously described raises the question to what would be considered an optimal design for clinical trials done in RTT patients where the patient population is small. Clinical heterogeneity seen in RTT patients could play a significant role and others have used alternative clinical trial designs such as the modified stepped wedge approach to manage the variability in the patient population [133]. In some cases, the design of the trials could be masking the improvements in some core features of RTT symptomatology from being revealed. Another significant problem with trials in rare diseases is the mathematical problem of increased variability arising from multiple evaluators (researchers and clinicians) because only a few subjects are available from each site participating in the trial. One strategy to deal with this could be that a central research site takes the responsibility for evaluating all the outcomes in a blinded manner, using digital health strategies or building in limited visits to the main site for critical time points when outcomes evaluation is essential. Reduction in evaluator variability will improve the likelihood of identifying true differences between placebo and the experimental drug.

Exaggerated placebo responses can also mask the actual effect especially in the design of double-blind randomised trials. A large placebo-like response was observed in children with autism spectrum disorders, whereby caregivers reported a significant reduction in problem behaviours in the absence of treatment [136]. Moreover, one study done in RTT patients has shown a placebo effect of more than 60% [118]. Whilst wishing not to speculate on how to reduce the magnitude of the placebo effect, newer trial designs such as the two-by-two blind trial design [137] or a result dependent randomisation algorithm [138] might be adopted to mitigate such issues.

Nevertheless, these studies have paved the way for pivotal trials to be performed and have opened up other important avenues for consideration such as the use of stratification biomarkers for patients entering into clinical trials. Some progress has been made in the area, for example, the use of the frontal band asymmetry index [124] and visual evoked potentials [139] to assess anxiety/depression and brain function respectively. Biometric guided therapy that monitors the autonomic component of EBAD in RTT patients can also be used to guide the stratification of patients entering clinical trials and will be discussed in the next section.

### Longitudinal monitoring of EBAD across the lifespan in patients with RTT

We have shown previously that biometric guided therapy can be used as a multimodal biomarker together with behavioural and emotional indices to manage EBAD in patients with RTT and other neurodevelopmental disorders [91, 92]. By objectively measuring the autonomic dysregulation, coupled with both observational and carer reported measures of behavioural and emotional dysregulation, EBAD could be considered as a tangible target for clinical trials. It would be useful in guiding researchers in improving inclusion criteria and hence the stratification of patients entering clinical trials. The inclusion of autonomic parameters has already led to an improved classification of patients with RTT allowing for a better prognosis [140]. Whilst this area of research in RTT is emerging, it is hoped that the development of new measures using the web-based HealthTracker^TM^ health monitoring and analytics system  [141] coupled with improved classification techniques [140] will boost the predictive power of stratification and allow researchers to capture clinically meaningful change of RTT symptomatology across the lifespan. Since RTT is not a homogeneous disorder but rather characterised by a nebulous developmental heterogeneity, capturing change across the lifespan would be crucial when designing stratification strategies for trials. To improve the clinical utility of such studies at least three time-points would be required so that EBAD could be tracked and have prognostic value. The longitudinal trajectory of EBAD has never been evaluated and in this view, one could have a model whereby EBAD could be tracked from childhood to adolescence where the neuro-anatomical and neuro-physiological profiles of RTT would be different. This may seem as an ambitious undertaking; however, given the multifaceted nature of RTT, new tangible outcome measures would be of benefit in terms of how we evaluate the efficacy of drugs in future clinical trials in RTT patients.

## Conclusions

The take home message from this review is that clinical trials undertaken in patients with RTT have met with limited success. None of the studies so far have specifically assessed EBAD and those that have explored autonomic parameters showed a mixed profile on the efficacy of interventions. Some recent clinical trials have shown promise in ameliorating the core features in RTT with improvements in autonomic parameters. However, given the loss of *MeCP2* capacity in fine-tuning neuronal brain development during early life and therefore triggering changes in the epigenetic states of genes potentially lasting across the life-span [142], it is very likely that single drug strategies may not be sufficient. Given the state of dysregulation of multiple overlapping physiological systems in EBAD, it is probable that there is a need for a symptom-based approach where different medications will be needed to manage different symptoms that impair subjects with RTT, thus requiring a truly personalized approach to treatment.

## Final remarks and the way forward

RTT is a complex disorder and given its mutational profile; treatment has been a real clinical challenge, however, this in an exciting time for the RTT field. A concerted effort is ongoing to optimise clinical trial design and yet due to the variability in the velocity of clinical presentation, it is vital to identify unifying features in RTT so that they can be measured across clinical trials. While no outcome measure will be perfect, it would be of benefit to create better instruments for intervention studies in RTT. The development of the comprehensive web based HealthTracker™ Rett Evaluation of Symptoms and Treatments (REST) questionnaire [141] along with objective measures such as biometric data is an important step forward in this area. These measures can be used to manage EBAD and can help to define subgroups of patients which will guide researchers to identify at baseline which group of patients might respond better to a particular treatment in the clinic or a drug in a clinical trial. Machine learning might also be a useful foil to consider. Recently, such an approach was used to predict motor progression in patients with Parkinson’s disease thereby offering up new ways to increase the cost-effectiveness and efficiency of clinical trial design [143]. This approach is likely to have clinical value in RTT as recently machine learning was shown to be able to overcome inter-subject and inter-trial variability when classifying respiratory disturbances in patients with RTT [144]. In a rare disease population, it may also be appropriate to consider personalised strategies to measure the effectiveness of medication by using web-based systems such as the HealthTracker^TM^ [93].

In summary, a multi-modal holistic approach is warranted to render meaningful clinical improvement in patients with RTT. The implementation of new outcome measures such as those focusing on EBAD will provide researchers with the opportunity to target a number of systems and aid in the development of better clinical trial designs.
